# MTP-STG: Spatio-Temporal Graph Transformer Networks for Multiple Future Trajectory Prediction in Crowds

**DOI:** 10.3390/s25247466

**Published:** 2025-12-08

**Authors:** Zichen Zhang, Xingwen Cao, Yi Song, Wenjie Gong, Liyu Zhang, Yanzhen Zhang, Yingxiang Li, Haoran Zhang

**Affiliations:** 1 School of Urban Planning and Design, Peking University, Shenzhen 518055, Chinaxingwen_cao@pku.edu.cn (X.C.);; 2 Institute of Biomedical and Health Engineering, Shenzhen Institutes of Advanced Technology, Chinese Academy of Sciences, Shenzhen 518055, China; 3 College of Forestry, Beijing Forestry University, Beijing 100083, China

**Keywords:** deep learning, trajectory prediction, object tracking, Transformer, graph neural networks

## Abstract

Predicting multiple future pedestrian trajectories is a challenging task for real-world applications like autonomous driving and robotic motion planning. Existing methods primarily focus on immediate spatial interactions among pedestrians, often overlooking the impact of distant spatial environments on their future trajectory choices. Additionally, aligning trajectory smoothness and temporal consistency remains challenging. We propose a multimodal trajectory prediction model that utilizes spatio-temporal graphical attention networks for crowd scenarios. Our method begins by generating simulated multiview pedestrian trajectory data using CARLA. It then combines original and selected multiview trajectories using a convex function to create augmented adversarial trajectories. This is followed by encoding pedestrian historical data with a multitarget detection and tracking algorithm. Using the augmented trajectories and encoded historical information as inputs, our spatio-temporal graph Transformer models scaled spatial interactions among pedestrians. We also integrate a trajectory smoothing method with a Memory Storage Module to predict multiple future paths based on historical crowd movement patterns. Extensive experiments demonstrate that our proposed MTP-STG model achieves state-of-the-art performance in predicting multiple future trajectories in crowds.

## 1. Introduction

In research on intelligent transportation systems and autonomous driving technology, multitarget pedestrian tracking and multifuture trajectory prediction are key areas of focus. The aim of multitarget pedestrian tracking is to monitor the positions and movements of multiple pedestrians within a video sequence. In real-world scenarios, pedestrian movements can be influenced by various factors, including the behavior of other pedestrians, traffic rules, and environmental conditions. Researchers have developed numerous methods that enhance the performance of multitarget pedestrian tracking to a certain extent [1,2]. Predicting pedestrian trajectories is crucial in computer vision, especially for applications in autonomous driving [3,4], surveillance [5,6], and human–robot interaction [7]. Although single-future trajectory prediction has been extensively studied, the inherent uncertainty and multimodality of human behavior necessitate exploring multifuture trajectory prediction. This task aims to generate multiple plausible future paths for pedestrians, reflecting the diverse possibilities of human movement in complex environments. Recent advancements in deep learning and graph-based methods have significantly improved the accuracy and robustness of these predictions. However, challenges remain in accurately modeling the spatial-temporal dependencies and social interactions among pedestrians.

Pedestrian trajectory prediction has evolved from deterministic methods to probabilistic and multimodal frameworks. Initial approaches like Social LSTM [8] and Social GAN [9] modeled social interactions using recurrent neural networks (RNNs) and generative adversarial networks (GANs). However, these methods could only predict a single future trajectory, overlooking the multimodality of human behavior. Recent studies have introduced multifuture prediction frameworks utilizing memory modules, graph-based spatial Transformers, and probabilistic models to better capture the diversity of pedestrian movements. A key challenge in multifuture trajectory prediction is accurately modeling spatial-temporal dependencies. Pedestrian movements are influenced by their past trajectories and their interactions with other pedestrians and the environment. Graph-based methods like STGAT [10] and Trajectron++ [11] show promise in capturing these dependencies by representing pedestrians as nodes and their interactions as edges in a graph. However, these methods struggle with long-term dependencies and the dynamic nature of social interactions. Evaluating multifuture predictions presents another challenge. Traditional metrics, such as Average Displacement Error (ADE) and Final Displacement Error (FDE), fall short in assessing the diversity and plausibility of multiple predicted trajectories. Recent studies have introduced metrics like $minADE_{k}$ and $minFDE_{k}$ [12] and PTU [13], to more effectively evaluate multifuture prediction models.

Recently, several innovative approaches have been developed to address the challenges of multifuture trajectory prediction. For example, the SocialCircle model [14] uses an angle-based social interaction representation, inspired by marine animals’ echolocation, to dynamically model pedestrian interactions. This approach divides the surrounding space into angular partitions and aggregates interaction data within each, offering a detailed representation of social dynamics. Another significant advancement is the Forking Paths Dataset [15], which fills the gap in diverse and realistic datasets for multifuture prediction. Created with a 3D simulator, this dataset enables human annotators to generate multiple plausible future trajectories, serving as a robust benchmark for evaluating multifuture prediction models. Memory-based methods have also become popular in this area. The SHENet framework [16] uses a memory bank to store historical group trajectories and a cross-modal Transformer to refine predictions based on individual-environment interactions. This method excels in constrained environments where human movements follow certain patterns. Additionally, integrating probabilistic models like the Gaussian Mixture Model (GMM) and Variational Autoencoder (VAE) has enabled the creation of diverse and plausible future trajectories. For instance, Multiverse [15] introduces a multidecoder framework that predicts both coarse-grained and fine-grained future locations, enhanced with semantic segmentation features to boost prediction accuracy.

However, despite these advancements, existing methods face three critical limitations. First, graph-based approaches typically rely on single-scale interaction graphs, often overlooking distant environmental context that influences long-term planning. Second, generative models excel in diversity but often lack explicit mechanisms to ensure temporal consistency, occasionally resulting in “jittery” paths. Third, most prediction models operate on the assumption of perfect ground-truth inputs, lacking robustness to detection noise. To overcome these challenges, we propose MTP-STG, a unified framework that specifically incorporates multiscale modeling for distant context and a memory mechanism to enforce temporal consistency, ensuring robustness in end-to-end tracking scenarios.

Multiobject TRACKING and multifuture trajectory prediction are inherently interconnected, relying on current and historical trajectory information provided by multitarget pedestrian tracking for pedestrians. Using a multiobjective tracker, the precise location and motion state of each pedestrian are detected in real time, enhancing the performance of multifuture trajectory prediction and vice versa. In this paper, we introduce MTP-STG, an integrated framework for multipedestrian tracking and multifuture trajectory prediction. Our model initially uses the MOTR detector for multitarget pedestrian tracking in video sequences to gather trajectory data for each pedestrian; this data is then fed into the spatio-temporal graph Transformer to predict the probability distribution of their multiple future trajectories. This integrated approach enables our model to simultaneously track multiple pedestrians and predict their future trajectories, providing a comprehensive understanding of pedestrian dynamics. The main contributions of this work are summarized as follows:

1. We integrate multitarget tracking and multifuture trajectory prediction into a unified framework for seamless end-to-end detection and prediction, effectively handling noisy inputs in real-world scenarios.
2. We develop a spatio-temporal graph Transformer incorporating a Multiscale Grid Graph structure. This design allows the model to simultaneously capture local social interactions and distant environmental semantics.
3. We introduce a Memory Storage Module within the trajectory generator. By retrieving and conditioning on historical embeddings, this module ensures the smoothness and temporal consistency of the diverse predicted trajectories.

## 2. Related Works

### 2.1. Multiobject Tracking Models

Multiobject Tracking (MOT) is designed to automatically detect and track multiple objects within a video sequence. Recently, MOT research has made significant strides, broadly categorized into traditional and deep learning approaches. Traditional MOT methods include SORT [17], DeepSORT [18], and Tracktor [19]. These methods typically involve two phases: detection and data association. The detection phase identifies the target’s position in each frame using a detector, while the association phase employs heuristic rules or optimization algorithms to link targets across frames and form their trajectories. The advantage of these methods is their simplicity and efficiency. However, they rely heavily on detector performance and struggle with complex motion patterns and occlusions.

Deep-learning-based MOTs use neural networks to learn the appearance and motion characteristics of targets, improving model performance and robustness. This approach can be divided into two types: the first treats target detection and association as independent subtasks, exemplified by Track-RCNN [20], JDE [21], and FairMOT [22]. These methods leverage existing detection and Re-ID techniques but often overlook the spatio-temporal relationships between targets and still require heuristic post-processing. The second type handles target detection and association as a unified task, as seen in TransMOT [23], TransTrack [24], and TrackFormer [25]. These approaches, based on Transformers [26] or graph neural networks [27], model the spatio-temporal relationships between targets to achieve end-to-end MOT. In the field of MOT, attention mechanisms are now a crucial research tool. The advantage of this approach is that it explicitly learns the motion patterns and interdependencies of targets, reducing the need for subsequent processing. However, it requires more computational resources and training data.

### 2.2. Single-Future Trajectory Prediction

Pedestrian trajectory prediction has gained significant attention for its essential role in autonomous driving, motion tracking, and robotic navigation. Early methods used deterministic models to predict a single future trajectory based on past observations. A pioneering model, Social LSTM, employed long short-term memory (LSTM) networks to model social interactions among pedestrians, improving prediction accuracy in crowded settings [8]. Subsequently, Social GAN leveraged generative adversarial networks (GANs) to generate socially acceptable trajectories, accounting for the stochastic nature of human motion [9]. However, these methods focus mainly on single-future prediction, limiting their ability to capture the inherent uncertainty and variability of pedestrian paths. Later research expanded on these foundations to enhance prediction accuracy and robustness. For example, SoPhie incorporated an attentive GAN model to account for both social interactions and physical constraints [28]. Likewise, the SR-LSTM model optimized LSTM states for improved pedestrian trajectory prediction in dynamic environments [29]. Despite these advancements, accurately predicting multiple plausible future trajectories remains challenging, necessitating further research into more advanced methods.

Graph neural networks (GNNs) have proven to be powerful tools for modeling relational data and have been effectively applied to trajectory prediction tasks. A recursive social behavior graph (RSBG) based on graph convolutional networks (GCNs) was introduced to model social interactions among pedestrians, capturing dynamic behaviors influencing pedestrian movement [30]. This model highlights the potential of GNNs in understanding complex social interactions and improving trajectory prediction accuracy. The STGAT model utilizes a spatio-temporal graph attention network to capture both spatial and temporal dependencies in pedestrian movements [10]. This method employs attention mechanisms to emphasize relevant interactions, enhancing prediction accuracy in dynamic environments. Another notable advancement is Trajectron, a probabilistic multiagent trajectory model that utilizes dynamic spatio-temporal graphs to predict future paths [31]. This model focuses on multiagent interactions, making it particularly effective in crowded and complex scenarios. Further advancements include Social-STGCNN, which incorporates spatio-temporal graph convolutional neural networks (ST-GCNNs) to predict human trajectories while accounting for social and physical constraints [32]. This model seamlessly integrates social dynamics with physical movement patterns, improving prediction accuracy across different environments. Despite these developments, integrating GNNs with other advanced techniques, such as Transformers and probabilistic models, could further enhance diversity and temporal consistency in multifuture predictions.

### 2.3. Transformer-Based Models

Transformer architectures have become increasingly popular for their ability to process sequential data across various domains. Initially developed for natural language processing, Transformers have since been adapted for computer vision and trajectory prediction. Spatio-temporal Transformer networks utilize spatial and temporal attention mechanisms to model pedestrian trajectories, effectively capturing long-range dependencies and complex temporal patterns [33]. The DETR model showcased the potential of Transformers in vision tasks, motivating their adoption in trajectory prediction [34]. Its success in object detection underscores the versatility of Transformer architectures. Vision Transformers (ViT) further demonstrated the effectiveness of Transformers in processing visual data. Their self-attention mechanism enhances spatial relationship modeling and improves the accuracy of future movement predictions [35]. AgentFormer [36] employs agent-aware Transformers for socio-temporal multiagent forecasting, emphasizing interactions between agents and their environment. This model provides a comprehensive approach to multifuture trajectory prediction by integrating Transformers with advanced interaction modeling. Combining Transformers with GNNs and probabilistic frameworks presents promising research directions, potentially enhancing accuracy, diversity, and computational efficiency in trajectory prediction. Different from previous methods [10,32] that rely on single-scale graphs to model local neighbors, our approach introduces a Multiscale Grid Graph structure. This allows the Transformer to attend to both fine-grained local interactions and coarse-grained distant environmental contexts simultaneously, providing a more holistic view of the scene.

### 2.4. Multifuture Trajectory Prediction

Acknowledging the limitations of single-future models, recent research has increasingly focused on predicting multiple plausible future trajectories. The Multiverse model proposed a two-stage probabilistic framework to generate diverse future trajectories, marking a significant advancement in the field [15]. However, ensuring temporal consistency across multiple predictions remains a challenge. Methods employing determinantal point processes (DPPs) enhance the diversity of predicted trajectories but often struggle to fully capture spatial interactions [37]. Beyond probabilistic models, researchers have explored several deep-learning-based approaches. The MultiPath model generates multiple probabilistic anchor trajectory hypotheses for behavior prediction, improving its capability to forecast diverse future scenarios [38]. Likewise, the Social-WaGDAT model utilizes a Wasserstein graph double-attention network to enhance interaction-aware trajectory prediction [39]. While effective, these methods can be computationally demanding due to their complexity. Another notable contribution is the approach in [40], which regularizes neural networks for trajectory prediction through an inverse reinforcement learning framework. This method emphasizes learning socially aware motion representations, ensuring predicted trajectories are diverse and contextually relevant. Despite these advancements, balancing prediction accuracy, diversity, and computational efficiency remains an ongoing research challenge. While methods like Multiverse [15] and SimAug [12] successfully generate diverse hypotheses, they do not explicitly model the temporal consistency of latent states over long horizons. Our MTP-STG addresses this by incorporating a Memory Storage Module. Unlike standard recurrence, our memory module allows the model to retrieve and condition on past historical embeddings, ensuring that the generated diverse trajectories remain smooth and temporally consistent. Most recently, diffusion-based approaches have set new benchmarks in trajectory prediction. For instance, SingularTrajectory [41] proposes a universal diffusion framework that iteratively denoises trajectories to achieve high precision across various domains. While these methods achieve state-of-the-art accuracy, their iterative sampling process often incurs high computational latency, limiting their applicability in real-time end-to-end tracking systems. In contrast, our MTP-STG focuses on an efficient one-shot prediction paradigm that balances accuracy and speed for crowd monitoring.

## 3. Proposed Method

### 3.1. Problem Description

The pedestrian trajectory prediction task is generally defined as there are n pedestrians in the geographic space, and given the time $t=1,\;2,\ldots ,\;T_{obs}$ moments, the trajectory spatial coordinates of the group of pedestrians in the geographic space at different moments $X=X_{1},\;X_{2},\ldots ,\;X_{n}\in R^{2}$, and predicts the future trajectory coordinates of all of them $\hat{X}={\hat{X}}_{1},\;{\hat{X}}_{2},\ldots ,\;{\hat{X}}_{n}\in R^{2}$ at $t=T_{obs}+1,\ldots ,\;T_{final}$ moments. The true spatial coordinates of each pedestrian i=1, 2,…, n in the geographic scene at the moment $t=1,\;2,\ldots ,\;T_{obs}$ are further represented as $X_{i}^{t}=\left(x_{i}^{t},y_{i}^{t}\right)\in R^{2},\;t=1,\;2,\ldots ,\;T_{obs},\;\forall_{i}\in i=1,\;2,\ldots ,\;n$. The predicted coordinates of the pedestrian group at the moment $t=T_{obs}+1,\ldots ,\;T_{final}$ are ${\hat{X}}_{i}^{t}=\left({\hat{x}}_{i}^{t},{\hat{y}}_{i}^{t}\right)\in R^{2},\;t=T_{obs},\ldots ,\;T_{final},\;\forall_{i}\in i=1,\;2,\ldots ,\;n$. The predicted trajectory duration is $T_{pred}=T_{final}-T_{obs}$. Our goal is to learn a model *f* that takes as input each pedestrian’s trajectory $X_{i}^{t}$ at $1∼T_{obs}$, learns the weight parameter $W^{\prime }$, and predicts each pedestrian’s trajectory ${\hat{X}}_{i}^{t}=f\left(X_{i}^{t},W^{\prime }\right)$ at moments $T_{obs}+1∼T_{final}$.

### 3.2. Overall Framework

**Figure 1:**
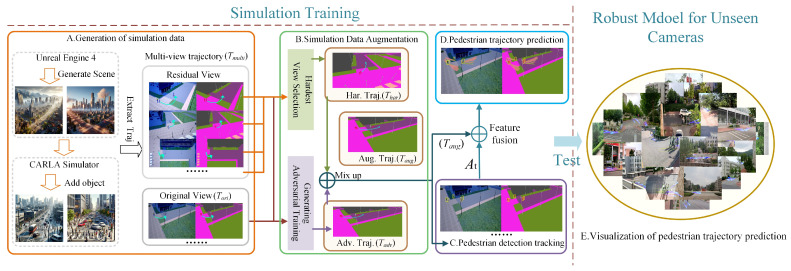
Overview of our method for MTP-STG, which is trained in simulation and tested on real unknown videos. Each training trajectory is extracted from the CARLA simulator and represented with multiview semantic segmentation features. MTP-STG mixes features from the hardest camera view with adversarial features from the original view to form an augmented adversarial trajectory. MOTR performs detection tracking on the scene crowd and records historical trajectory information. The trajectory prediction network outputs multiple future trajectory probability distributions for pedestrians.

The Pedestrian Multifuture Trajectory Prediction (MTP-STG) framework, based on the Spatio-Temporal Graph Attention Mechanism, consists of four modules, as illustrated in Figure 1.

A. Simulation Data Generation Module: Pedestrian training trajectories from different viewpoints are generated using the CARLA [42] simulator and represented as a multiview semantic segmentation feature map.
B. Simulation Data Enhancement Module: The most challenging trajectory $T_{har}$ is selected from a given set of multiview trajectories $T_{multi}$. Then, $T_{har}$ and $T_{adv}$ are combined using the Mixup [43] convex function to generate the enhanced trajectory $T_{aug}$.
C. Pedestrian Detection and Tracking Module: Pedestrian tracking and historical trajectory encoding are performed using the MOTR multitarget detection tracker.
D. Pedestrian Multifuture Trajectory Prediction Module: The Spatio-Temporal Graph Attention Mechanism is used as the backbone for trajectory prediction. The augmented trajectory $T_{aug}$ and historical trajectory encoding information $A_{t}$ serve as network inputs to achieve multifuture trajectory prediction for crowds in spatial environments.

### 3.3. Multiview Simulation Data and Augmentation

The multiview simulation trajectory training data is derived from the VIRAT/ ActEV [44,45] real-world dataset and is semi-automatically labeled using the CARLA [42] simulator. This dataset captures pedestrian trajectories from four different viewpoints, along with pedestrian detection frame annotations and scene semantic features. The generated simulated video trajectory sequences closely resemble real video sequences in terms of both pedestrian appearance and motion trajectories. We further define the simulation trajectory training data. At a given time $t=1,\;2,\ldots ,\;T_{obs}$, the trajectory coordinates of a group of pedestrians in the scene are represented as $X=X_{1},\;X_{2},\ldots ,\;X_{n}\in R^{2}$, which we denote as $\left(V_{1:n},\;L_{1:n}\right)$. Here, $\left(V_{1:n}\right)$ represents the consecutive video frames from 1 to *n*, and  $\left(L_{1:n}\right)$ represents the corresponding coordinate positions. For $t=T_{obs}+1,\ldots ,\;T_{final}$, the future trajectories of pedestrian crowds are denoted as $\hat{X}={\hat{X}}_{1},\;{\hat{X}}_{2},\ldots ,\;{\hat{X}}_{n}\in R^{2}$. These are defined as $\left(L_{n+1}|V_{1:n},\;L_{1:n}\right)$, representing the predicted trajectory coordinates of pedestrians over T−n frames, where $L_{n+1:T}$ is given by:

(1) $$L_{n+1:T}=p_{n+1}\left(x_{n+1},\;y_{n+1}\right),\ldots ,\;p_{T}\left(x_{T},\;y_{T}\right)$$

**Figure 2:**
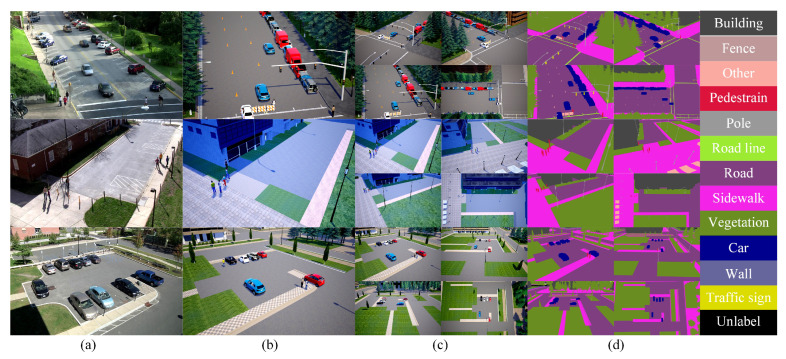
Visualization of simulation data. (**a**) Real scene from the VIRAT/ActEV dataset. (**b**) Reconstructed scene from the VIRAT/ActEV dataset using CARLA and Unreal Engine 4. (**c**) The corresponding scene visualized from four different viewpoints: three 45° oblique views and one 90° overhead view. (**d**) Semantic segmentation of the real scene into C=13 categories, including sidewalks, roads, vehicles, and pedestrians.

Let the complete training trajectory under the original view-point trajectory $T_{ori}$ in the training data be $\left(V_{1:T},\;L_{1:T}\right)$, due to the different coordinate representations of the same trajectory under the rest of the viewpoints, $L_{1:T}^{i}\neq L_{1:T}^{j},\;i\neq j$, using K to represent the different viewpoints of the same trajectory, which is expressed through Equation (2) as:

(2) $$K={\left\{\left(V_{1:T}^{i},\;L_{1:T}^{i}\right)\right\}}_{i=1}^{\left|K\right|}$$

for the set *S* of multiview trajectories $T_{multi}$, one trajectory at a time is selected from *S* and used as an anchor point to search for the most inconsistent viewpoints with what the model has learned, which we refer to as the hardest-to-learn view trajectories in the text. Inspired by the classification loss function proposed in [46], we use it as a criterion for calculating the loss of a given viewpoint trajectory with respect to the hardest-to-learn views trajectory:

(3) $$j^{*}=\underset{j\in [1,|K|]}{\arg\;\max}\left\{L_{class}\left(V_{1:n}+\delta ,\;L_{n+1:T}^{j}\right)\right\}$$

where $j^{*}$ is denoted as the index of the viewpoint with the highest classification loss; $V_{1:n}$ is denoted as the scene semantic segmentation feature of the trajectory frame; $L_{n+1:T}^{j}$ is denoted as the future location label of the j-th viewpoint; δ is the random perturbation of the input feature; and $L_{cls}$ is the loss function for location classification used by the GATRNN adopted by the Trajectory Prediction Network module. For the original trajectory perspective trajectory $T_{ori}$, the adversarial trajectory $V_{1:n}^{adv}$ is generated using the Targeted-FGSM [47] network, computed as:

(4) $$V_{1:n}^{adv}=V_{1:n}-\epsilon \cdot \mathrm{sign}\left({▽}_{V_{1:n}}L_{cls}\left(V_{1:n}+\delta ,\;L_{n+1:T}^{j^{*}}\right)\right)$$

where ϵ is denoted as a hyperparameter, and the use of the adversarial learning method enables the model to select the most difficult to learn view to predict the future trajectory position of the pedestrian in a given multiview trajectory, instead of predicting the trajectory position in the original view. The random perturbation δ is added to reduce the error caused by the uncertainty of the data itself. For better stability of the MTP-STG model for low-resolution visual features and different scene viewpoint transitions, the effect of subtle noise generated by different lighting conditions, scene textures, and camera sensors in the generated training data is reduced. We use the Mixup [43] convex function to mix the hardest-to-learn view trajectory $T_{har}$ and the generative adversarial trajectory $T_{adv}$ to generate the augmented trajectory $\left(V_{1:n}^{aug},\;L_{1:n}^{aug}\right)$, which is calculated by the following:

(5) $$V_{1:n}^{aug}=\lambda \cdot V_{1:n}^{adv}+\left(1-\lambda \right)\cdot V_{1:n}^{j^{*}}$$

(6) $$L_{n+1:T}^{aug}=\left\{p_{n+1}^{aug},\ldots ,\;p_{T}^{aug}\right\}$$

(7) $$p_{T}^{aug}=\lambda \cdot \text{one-hot}\left(p_{t}\right)+\left(1-\lambda \right)\cdot \text{one-hot}\left(p_{t}^{j^{*}}\right),\;\forall t\in \left[n+1,\;T\right]$$

where λ is derived from the beta(α, α) distribution controlled by the hyperparameter α; $p_{t}$ is the true trajectory coordinate in Equation (2) representing the original viewpoint; and the one-hot function maps the x-y 2D coordinate position projection onto a predefined grid in the trajectory prediction network module. In addition, we train the Deeplabv3 [48] semantic segmentation model on the Cityscapes dataset for extracting real scene semantic features. In order to minimize the difference between real and simulated video frames, we represent all trajectories $\left(V_{1:T}^{\left(i\right)},\;L_{1:T}^{\left(i\right)}\right)$ as semantic segmentation sequence features and locations, $V_{t}^{\left(i\right)}$ denotes the semantic segmentation features of the scene for the trajectory under the i-th viewpoint at the moment of time *t*, and $L_{t}^{\left(i\right)}$ denotes the location coordinate values under the i-th viewpoint sequence. Figure 2 shows the generated multiview simulation video pedestrian trajectory data visualized in different scene view. The core intuition behind using Adversarial Mixup is to enforce view-invariance. By mixing features from the “hardest-to-learn” view (which yields the highest loss) with the original view, and adding random perturbations δ, we simulate challenging conditions such as camera noise, varying lighting, or poor sensor quality. This forces the model to learn robust features that are invariant to specific camera angles. Regarding hyperparameters, the mixing coefficient α in the Beta distribution controls the intensity of interpolation. We empirically set α=0.2 based on a grid search, finding that this value provides sufficient diversity without destroying the semantic integrity of the pedestrian features. Similarly, the perturbation magnitude ϵ=0.1 was chosen to balance robustness and training stability.

### 3.4. Crowd Detection Tracking Module

**Figure 3:**
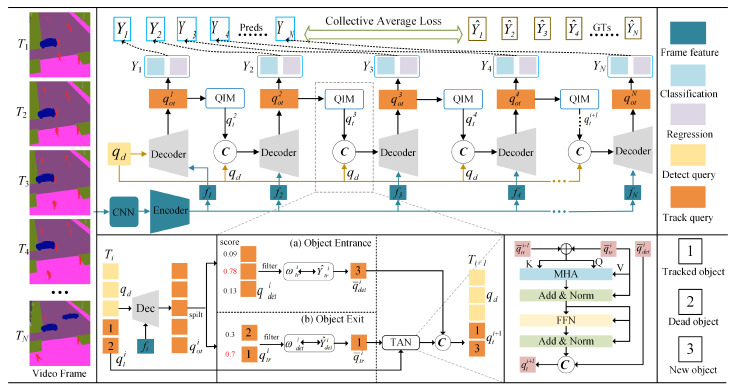
The overall architecture of the MOTR encoder represents the convolutional neural network backbone and the Transformer encoder that extracts the features of each image frame. A cascade of detection query $q_{d}$ and tracking query $q_{tr}$ is fed into the decoder (Dec) to generate hidden states. The hidden state is utilized to generate the prediction $\hat{Y}$ for newborn and tracked objects. The QIM module takes the hidden state as input and generates the tracking query for the subsequent frame.

Unlike previous studies [8,9], which used the final hidden layer state of LSTM to model surrounding pedestrian information or abstract pedestrians as coordinate points in space, we employ the MOTR [49] network for crowd detection and tracking. Figure 3 presents a schematic diagram of crowd detection and tracking. In this paper, the simulated crowd trajectory video stream is modeled as a continuous sequence of images. Each frame is processed using a convolutional neural network backbone (ResNet50) and a Transformer encoder to extract image features. The detection query, denoted as $q_{d}$, consists of fixed-length queries designed to identify newly emerging pedestrians in a sequence of simulated crowd trajectory video frames. The tracking query, denoted as $q_{tr}$, represents a continuously tracked crowd object in a sequence of simulated crowd trajectory video frames. It consists of dynamically updated queries. For consecutive video frames, the concatenation of the variable tracking query $q_{tr}$ from the previous frame with the fixed detection query $q_{d}$, along with extracted image features, is fed into the Transformer-based decoder to generate the hidden state of the predicted crowd bounding box. This output is then fed into the Query Interaction Module to generate the trajectory query for the next frame. In this paper, all pedestrian bounding box predictions in the video stream are aggregated into the collection $\left\{{\hat{Y}}_{1},\;{\hat{Y}}_{2},\ldots ,\;{\hat{Y}}_{N}\right\}$.

The Query Interaction Module consists of two components: a pedestrian entry and exit mechanism and the Temporal Aggregation Network (TAN). To handle pedestrian appearance and disappearance in video sequences, we propose a crowd trajectory-aware label assignment method, Tracklet-Aware Label Assignment (TALA). For the detection query $q_{d}$, we follow the approach in MOTR and modify the assignment strategy to newborn-only, which performs bipartite graph matching exclusively among newly appeared objects. For the tracking query $q_{tr}$, we apply a goal-consistent assignment strategy, where the tracking query follows the assignment result from the previous frame and bypasses bipartite graph matching. Specifically, we define the tracking query prediction as ${\hat{Y}}_{tr}$ and the detection query prediction as ${\hat{Y}}_{det}$. Here, $Y_{new}$ represents the newly detected pedestrian object. The label assignment results for the tracking and detection queries are denoted as $\omega_{tr}$ and $\omega_{det}$, respectively. For frame *i*, the label assignment for the detection query is determined by performing bipartite graph matching between the detection query and the newly appeared pedestrian target, as follows:

(8) $$\omega_{det}^{i}=\underset{\omega_{det}^{i}\in \Omega_{i}}{\arg\;\min}\;\;\ell \left({\hat{Y}}_{det|\omega_{det}^{i}}^{i},\;Y_{new}^{i}\right)$$

In this context, *ℓ* denotes the matching function as defined in DETR [34], while $\Omega_{i}$ represents the space of all possible bipartite matches between the detection query $q_{d}$ and the newly detected pedestrian target. For the label assignment of the tracking query $q_{tr}$, we incorporate the assignments of both the newly detected pedestrian object and the tracked object from the previous frame, specifically for i>1:

(9) $$\omega_{tr}^{i}=\omega_{tr}^{i-1}\cup \omega_{det}^{i-1}$$

Since there are no trace objects in the first frame (i=1), the trace query $\omega_{tr}^{1}$ assignment is represented as an empty set ∅. However, for consecutive frames (i>1), the trace query assignment $\omega_{tr}^{i}$ is obtained by concatenating the previous trace query assignment $\omega_{tr}^{i-1}$ with the assignment of the new object $\omega_{det}^{i-1}$.

In the crowd entry mechanism, when a new pedestrian target appears in the video stream, its corresponding hidden state is updated according to the assignment rule defined in Equation (8). If a matched pedestrian target is no longer present in the scene, or if the IoU threshold score between the predicted bounding box and the target falls below 0.8, the current hidden state of the pedestrian target is discarded and terminated. This leads to the disappearance of these pedestrian targets in the current video frame, where their hidden states are filtered, and only the remaining hidden states are retained. In the crowd exit mechanism, the disappearance of both newly detected pedestrian targets and previously tracked pedestrian targets is determined based on their classification scores. For the detection query $q_{d}$, predictions with classification scores exceeding the entry threshold $\tau_{en}$ are retained, while other hidden states are discarded.

For the tracking query $q_{tr}$, predictions with classification scores below the exit threshold $\tau_{ex}$ across consecutive *M* frames are discarded, while other hidden states are retained. A Temporal Aggregation Network (TAN) is incorporated into the QIM module to enhance the modeling of temporal relationships within crowd trajectories and provide contextual a priori knowledge for tracking targets. The TAN, an enhanced Transformer decoder layer, receives the last frame of the tracking query $q_{tr}^{i}$ and the filtered hidden state as inputs to the Multihead Attention (MHA) module. Following the MHA, a feed-forward network (FFN) links the result with the hidden state of the new object to produce the trajectory query set $q_{tr}^{i}$ for the subsequent frame. At the conclusion of the detection and tracking process, for each input frame $f_{H\times W}^{n}$ at time *t*, a matrix $A_{t}$, as defined in Equation (10), contains the positions of the *n* detected pedestrians within the sequence frame grid $R^{H\times W}$.

(10) $$A_{t}=\left\{p_{\left(x_{n},y_{n}\right)}^{t}|x_{n}\in W,\;y_{n}\in H\right\}$$

### 3.5. Spatio-Temporal Graph Transformer Networks

**Figure 4:**
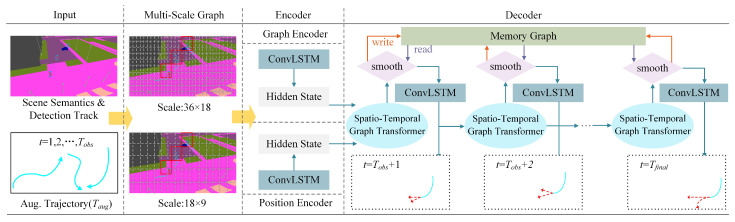
Overview of the Spatio-Temporal Graph Transformer Network. The graph encoder and the position encoder encode node-level and coordinate-level features, respectively, processed by a multiscale graph. At each decoding time step, our proposed Spatio-Temporal Graph Transformer predicts the next potential neighboring positions. A Memory Storage Module smooths and corrects trajectories that violate temporal consistency.

The comprehensive structure of the Pedestrian Multifuture Trajectory Prediction network is depicted in Figure 4. Inputs to the Multiscale Grid Graph include the augmented trajectory $T_{aug}$, scene semantics *S*, and pedestrian coding information $A_{t}$. These inputs are processed and relayed to the spatio-temporal encoding–decoding network, which generates the pedestrian multifuture trajectory. The encoder comprises a graph encoder and a position encoder, which, respectively, encode the node-level and coordinate-level features of the processed multiscale graph. At each time step, the spatio-temporal graph decoder aggregates the information and generates a probability distribution of the pedestrians’ locations in the subsequent time step using an LSTM cell. The memory map facilitates smooth prediction by reading and writing to the decoded trajectory memory map, which contains comprehensive temporal information.

#### 3.5.1. Multiscale Grid Graph and Sequence Encoder

We partition the video frame into multiple 2D regular grids, forming a graph G∈(V, E), with nodes *V* and edges *E*. Each grid cell, defined as a node v∈V, connects to its neighbors via undirected edges e∈E. Each grid cell establishes connections with its horizontal, vertical, and diagonal neighbors. Specifically, we design two distinct grid resolutions to capture different levels of spatial granularity. The coarse grid (18×9) is designed to capture global environmental context, such as distant road layouts and large static obstacles, allowing the model to plan long-term destinations. In contrast, the fine grid (36×18) focuses on local social interactions and precise pedestrian localization, enabling the model to handle immediate collision avoidance. By aggregating features from both scales, the model achieves a balance between global navigation and local safety. By leveraging multiscale graphs, the MTP-STG model adapts more effectively to varied information levels and makes comprehensive decisions based on the surrounding pedestrian environments. At each graph scale, the sequence encoder computes scene representations using the pedestrian’s augmented trajectory $T_{aug}$ and coded information $A_{t}$. The graph encoder encodes the node-level features of the grid cell index $Y_{t}\in G^{H\times W}$, corresponding to the current location. To enhance the model’s robustness against low-resolution visual features and varied viewpoint transitions, we employ the Deeplab v3+ semantic segmentation model [48], maintaining its weight parameters unchanged to encode each video frame $V_{t}$ into a semantic segmentation feature vector $S_{t}^{G}$ of size H×W×C. Pedestrian spatial features are computed using ConvLSTM [50] as follows:

(11) $$\begin{array}{cc} \; & H{\left(\right)}_{t}^{G}=ConvLSTM(\text{one-hot}\left(\mathrm{idx}{\left(x_{t},y_{t}\right)}^{G}\right)⊙\\ \; & \left(W_{e}*S_{t}^{G},H{\left(\right)}_{t-1}^{G}\right) \end{array}$$

where ⊙ is the product between elements, $W_{e}$ is the sequence encoder learnable weight parameter, the idx(·) function converts the pedestrian coordinates $\left(x_{t},\;y_{t}\right)$ to cell $G^{H\times W}$ indexes in a 2D grid, and the one-hot function projects the cell indexes to their corresponding positions on a map of size H×W scale according to their spatial positions. The position encoder is responsible for encoding the offsets of the center of the area covered by the node, calculated using Equation (12):

(12) $$H{\left(l\right)}_{t}^{G}=ConvLSTM\left(l_{t}^{G}\left({x_{t}^{\prime }}^{G},\;{y_{t}^{\prime }}^{G}\right),\;H{\left(l\right)}_{t-1}^{G}\right)$$

where $l_{t}^{G}\left({x_{t}^{\prime }}^{G},\;{y_{t}^{\prime }}^{G}\right)$ is calculated $\left(x_{t},\;y_{t}\right)-C\left(\mathrm{idx}{\left(x_{t},\;y_{t}\right)}^{G}\right)$, and the function C(·) retrieves the center coordinates of the indexed cell. $H{\left(\right)}_{t}^{G}$ and $H{\left(l\right)}_{t}^{G}$, respectively, represent the hidden states of the graph encoder and the position encoder for the 2D grid G at time *t*. Since both the encoder and the decoder subsequently process these two hidden states in the same manner, this paper treats the last hidden layer state of the sequence encoder as the final spatio-temporal state $H_{t}^{G}\in R^{H\times W\times d_{enc}}$ of pedestrians in the geographic scene. $d_{enc}$ is the size of the last hidden layer state. We also encode the spatio-temporal state $H_{t}^{G}$ and the semantic segmentation average ${\bar{S}}^{G}=\frac{1}{T_{obs}}\sum_{t=1}^{T_{obs}}S_{t}^{G}$ into a context vector $H_{T_{obs}}^{G}=\left[H_{t}^{G},\;{\bar{S}\;}^{G}\;\right]$, which is used to initialize the decoder.

#### 3.5.2. Spatio-Temporal Attention Transformer

We input the encoded hidden state $H_{T_{obs}}^{G}$ of the multiscale graph G into the spatio-temporal graph attention decoder, aggregating generative information from all node pairs through the graph attention mechanism. Subsequently, a group of node states is updated concurrently, denoting the pedestrian’s confidence state in the unit grid at future time *t* as $C_{t}\left(i\right)=p\left(Y_{t}=i\;|\;Y_{n:t-1},\;H_{T_{obs}}^{G}\right),\;\forall i\in G,\;t\in \left[n+1,\;T\right]$. For simplicity, this paper uses a single index i to denote a cell in the 2D grid $G^{H\times W}$. The confidence state $C_{t}$ is then updated and calculated via the hidden state $H_{t}^{C}$ of the ConvLSTM:

(13) $$\begin{array}{cc} \; & C_{t}=softmax\left(W_{c}*H_{t}^{C}\right)\in R^{H\times W}\\ \; & H_{t}^{C}=ConvLSTM\left(GAT\left(H_{t-1}^{C}\right),\;embed\left(C_{t-1}\right)\right) \end{array}$$

where $W_{c}$ represents the learnable weight parameters of the position decoder, which updates the hidden state $H_{t}^{C}$ using a 2D convolutional filter before applying softmax normalization. The confidence state $C_{t-1}$ is embedded into a three-dimensional tensor of size $H\times W\times d_{enc}$. The $GAT(H_{t-1}^{C})$ denotes a Graph Attention Network, where the graph structure G corresponds to the 2D grid within. Due to the graph attention mechanism incorporating both graph structure and spatial proximity advantages, it differentially focuses on various regions. Pedestrians exhibit strong spatial interactions with nearby areas. Objects in distant areas, such as vehicles and buildings, provide essential spatial cues for planning the future trajectory of pedestrians. Therefore, we compute the attention information $M_{a[i\to j]}^{G}$ from node $v_{i}^{G}$ to $v_{j}^{G}$ within the scale graph G, and the global information matrix $M_{g[i\to j]}^{G}$:

(14) $$\begin{array}{cc} \; & M_{a\left[i\to j\right]}^{G}=f_{V\left[i\right]}^{G}⊙\left(f_{Q\left[i\right]}^{G}\left|\right|f_{K\left[j\right]}^{G^{T}}\right)+{\bar{b}}^{G}\\ \; & M_{g\left[i\to j\right]}^{G}={\vec{h}}_{i}^{G}{\vec{h}}_{j}^{G^{T}} \end{array}$$

where $M_{a\left[i\to j\right]}^{G}$ is the information matrix from node $v_{i}$ to node $v_{j}$ within the scale graph G, utilized to learn the query matrix $f_{Q}^{G}$, key matrix $f_{K}^{G}$, and value matrix $f_{V}^{G}$ from the hidden state $H_{T_{obs}}^{G}$, || denotes the operation of concatenating the query matrix $f_{Q}^{G}$ with the transpose of the key matrix $f_{K}^{G}$. Subsequently, ⊙ involves an element-wise multiplication of the concatenated matrix with the value matrix, calculating the attention values for each node with respect to $v_{i}$ and $v_{j}$, and adding a bias ${\bar{b}}^{G}$. We perform an element-wise addition of $M_{a\left[i\to j\right]}^{G}$ with $M_{g\left[i\to j\right]}^{G}$, resulting in the total information matrix $M_{total\left[i\to j\right]}^{G}$ that is transmitted from node $v_{i}$ to $v_{j}$. To update the state of the next node, we define $h_{i}$ as the feature vector of the i-th grid cell in the hidden layer state $H_{t-1}^{C}$, and $\tilde{h_{i}}$ corresponds to the output of ${\tilde{H}}_{t-1}^{C}=GAT\left(H_{t-1}^{C}\right)\;\in \;R^{H\times W\times d_{enc}}$. $d_{dec}$ denote the size of the decoder hidden layer state, which can be computed using Equation (15):

(15) $${\tilde{h}}_{i}=\frac{1}{\left|N_{i}\right|}\sum_{j\in N_{i}^{G}}f_{e_{\left[i\to j\right]}}^{G}\left(\left[v_{i},\;v_{j}\right]\right)+h_{i}$$

where $N_{i}$ denotes the set of neighboring nodes of $v_{i}$ within the 2D grid G, $h_{i}$ represents the initial feature vector of the grid cell, and $f_{e_{\left[i\to j\right]}}^{G}$ is defined as attention-weight edge function that normalizes the total information matrix $M_{total\left[i\to j\right]}^{G}$ using the softmax function. The graph structure update function we implemented enables the model to diffuse probability mass between grid cells in a controlled manner. This model captures human relational dynamics, ensuring that as crowds navigate through a scene, they do not abruptly jump to distant locations. This foundational assumption or prior knowledge is embedded within the network’s convolutional architecture. Integrating the graph attention network enables dynamic adjustment of weights based on input, enhancing trajectory prediction significantly. Combining the above formulations, Equation (16) is used to represent the pedestrian’s position ${\hat{P}}_{t}^{G}$ in the unit grid at future time *t*.

(16) $${\hat{P}}_{t}^{G}=softmax\left(f_{c}\left(H_{T_{obs}}^{G},\;H_{t-1}^{C}\right)\right)\in R^{H\times W\times 1}$$

where $f_{c}$ denotes the GAT and $H_{t-1}^{C}$ represents the hidden layer state of the GAT. ${\hat{P}}_{t}^{G}$ denotes the position prediction at time *t*. For each node ${\hat{P}}_{t}^{G}\left(i\right)$ represents a probability when the input is sourced from the graph encoder. When the input derives from the position encoder, it represents a coordinate value offset from the center of node $v_{i}$. Although the pedestrian state context vector $H_{T_{obs}}^{G}$ incorporates semantic segmentation features ${\bar{S}\;}^{G}$ and facilitates trajectory prediction via an output heatmap, it lacks precise position estimation capabilities. To enhance the precision of predicted pedestrian trajectories, we introduce a second GAT decoder that predicts continuous offset increments within the $R^{2}$ region. These offset increments, represented by σ, detail the adjustments required at the center of the grid cell as predicted by the decoder. The refined position ${\hat{O}}_{t}$ is determined using Equation (17).

(17) $$\begin{array}{cc} \; & {\hat{O}}_{t}=MLP\left(f_{o}\left(H_{T_{obs}}^{G},H_{t}^{O}\right)\right)\;\in \;R^{H\times W\times 2}\\ \; & H_{t}^{O}=ConvLSTM\left(GAT\left(H_{t-1}^{O}\right),O_{t-1}\right)\in R^{H\times W\times d_{dec}} \end{array}$$

where MLP is used to embed each pedestrian’s positional coordinates into the vector representation of the $R^{2}$ grid; $f_{o}$ and $f_{c}$ are independent GAT modules; and $H_{t}^{o}$ is the hidden layer of $f_{o}$. The final predicted position of a pedestrian in the spatial scene is represented as ${\hat{L}}_{t}=Q_{m}+{\hat{O}}_{tm}$, where $m=arg\;max{\;\hat{p}}_{t}$ denotes the index of the selected grid cell, $Q_{m}\in R^{2}$ is the center of the selected grid cell, and ${\hat{O}}_{tm}\in R^{2}$ represents the offset increment from the center of the grid cell at time step *t*.

#### 3.5.3. Memory Storage Module

Although the spatio-temporal graph encoder–decoder in this study utilizes a self-attention mechanism to focus the model on the most probable areas, thereby enhancing the modeling of extended temporal sequences, it struggles with handling continuous time series data that require strong temporal consistency. Within the decoder, the hidden state at time step *t* heavily relies on the state from t−1 Furthermore, the current position is influenced by the hidden states from all preceding time steps. Relying solely on the most recent time step for future trajectory predictions can result in deviations from the initially predicted destination, as indicated by the earlier sequence of hidden states. To address this limitation, this paper introduces a straightforward, interpretable, and trainable external graphical memory module, denoted as $M_{1:T}$. This module serves two primary functions:

- Historical Context: It embeds historical trajectory information into a spatio-temporal graph Transformer, conditioning current predictions on past behaviors and enhancing temporal consistency.
- Smoothing Mechanism: It smooths trajectory embeddings through memory update operations, mitigating abrupt changes in predictions and ensuring trajectory coherence.

Firstly, the Memory Storage Module maintains an embedding $M_{t}\left(i\right)$ for each pedestrian *i* at every time step *t*, with dimensions equivalent to $h_{t}^{i}$. At each time step *t*, the spatio-temporal graph Transformer retrieves historical embeddings using the reading function $f_{read}$ from the memory graph. Specifically, for each pedestrian in the spatial scene, the function retrieves all prior embeddings from time steps $\left[1,\;t-1\right]:{\left\{\left\{{\tilde{h}}_{1}^{i},\;{\tilde{h}}_{2}^{i},\ldots ,\;{\tilde{h}}_{t-1}^{i}\right\}\right\}}_{i=1}^{N}=f_{read}\left(M\right)$. The reading function is defined as: $f_{read}\left(M\right)={\left\{M_{1}\left(i\right),M_{2}\left(i\right),\ldots ,\;M_{t-1}\left(i\right)\right\}}_{i=1}^{N}$. This function integrates the current graph embeddings ${\left\{h_{t}^{i}\right\}}_{i=1}^{N}$ with historical data, providing the Transformer with a comprehensive view of both past and present contexts. Upon processing the input, the spatio-temporal graph Transformer updates its output graph embeddings ${\left\{{{h^{\prime }}_{1}^{i},\;{{h^{\prime }}_{2}^{i}\;,\ldots ,\;h}^{\prime }}_{t}^{i}\right\}}_{i=1}^{N}$ into the graph memory using the writing function $f_{write}:M^{\prime }=f_{write}\left({\left\{{{h^{\prime }}_{1}^{i},\;{{h^{\prime }}_{2}^{i}\;,\ldots ,\;h}^{\prime }}_{t}^{i}\right\}}_{i=1}^{N},M\right)$. This ensures that the Memory Storage Module is updated with the latest embedding, thus achieving temporal smoothness and consistency. To provide a clearer understanding of the temporal update mechanism, the process of reading and writing embeddings in the Memory Storage Module is detailed in Algorithm 1.
**Algorithm 1** Memory Storage Module update process.**Require:** 
Graph Embeddings $H_{in}=\left\{h_{1},\ldots ,h_{T}\right\}$, History steps $T_{obs}$, Future steps $T_{pred}$**Ensure:** 
Predicted Trajectories $\hat{Y}$1:Initialize Memory Bank M←∅2:**for** t=1 to $T_{obs}+T_{pred}$ **do**3:   // 1. Read Operation (Eq. for $f_{read}$)4:   Retrieve historical context: $C_{hist}=f_{read}\left(M\right)$5:   // 2. Feature Integration6:   Integrate current embedding $h_{t}$ with context: $h_{t}^{\prime }=\mathrm{Transformer}\left(h_{t},\;C_{hist}\right)$7:   // 3. Trajectory Prediction8:   Predict position offset: ${\hat{p}}_{t}=\mathrm{Decoder}\left(h_{t}^{\prime }\right)$9:   // 4. Write Operation (Eq. for $f_{write}$)10:   Update memory with new state: $M=f_{write}\left(M,\;h_{t}^{\prime }\right)$11:   Append ${\hat{p}}_{t}$ to $\hat{Y}$12:**end for**13:**return** $\hat{Y}$

### 3.6. MTP-STG Model Loss Function

The total loss of the MTP-STG model comprises the collective average loss from crowd detection $L_{CAL}$ and the multimodal trajectory prediction loss $L_{PRE}$, formulated as $L_{total}={\lambda_{1}L}_{o}+\lambda_{2}L_{PRE}$. Here, the set $\left\{\lambda_{*}\right\}$ balances the collective average loss and the multimodal trajectory prediction loss. The MOTR crowd detector learns temporal variances directly from the data, rather than relying on manually crafted heuristic methods like the Kalman filter. Unlike previous methods, MOTR processes pedestrian video streams as input, facilitating the generation of training samples that capture distant object movements for temporal learning. Instead of computing the loss frame-by-frame, $L_{o}$ accumulates losses across multiple predictions $\hat{Y}={\left\{{\hat{Y}}_{i}\right\}}_{i=1}^{N}$. The loss for the entire video sequence is calculated based on the ground truth $Y={\left\{{\hat{Y}}_{i}\right\}}_{i=1}^{N}$ and the matching results $\omega ={\left\{\omega_{i}\right\}}_{i=1}^{N}$. $L_{o}$ represents the total loss across the entire video sequence, normalized by the number of objects.

(18) $$L_{o}\left({\hat{Y}|}_{\omega },Y\right)=\frac{\sum_{n=1}^{N}\left(L\left({\hat{Y}}_{tr|\omega_{tr}^{i}}^{i},Y_{tr}^{i}\right)+L\left({\hat{Y}}_{det|\omega_{det}^{i}}^{i},Y_{det}^{i}\right)\right)}{\sum_{n=1}^{N}\left(V_{i}\right)}$$

where $V_{i}=V_{tr}^{i}+V_{det}^{i}$ represents the total number of real pedestrians in frame *i*, where $V_{tr}^{i}$ and $V_{det}^{i}$ denote the number of tracked and newly detected objects in frame *i*, respectively. The loss for a single video frame L, is expressed a $L\left({\hat{Y}}_{i|\omega_{i}},Y_{i}\right)=\lambda_{cls}L_{cls}+\lambda_{l_{1}}L_{l_{1}}+\lambda_{giou}L_{giou}$. Here, $L_{cls}$ is the focal loss, $L_{l_{1}}$ represents the $L_{1}$ loss, and $L_{giou}$ is the generalized Intersection over Union (IoU) loss. The coefficients $\lambda_{cls}$, $\lambda_{l_{1}}$, and $\lambda_{giou}$ are the respective weighting factors.

The multimodal trajectory prediction loss $L_{PRE}$ comprises the cross-entropy loss $L_{c}^{G}$ from the graph encoder and the regression loss $L_{r}^{G}$ from the location encoder. To leverage benefits from multiscale graphs G, this study utilizes a multiscale discriminator and calculates losses at two scales, denoted as Scales∈[36×18,18×9], and expressed as $L_{PRE}=\sum_{G\in Scales}\alpha_{1}L_{c}^{G}+\beta_{1}L_{r}^{G}$. Each graph scale G at each time t considers the true output as $P_{i}^{G}\left(t\right)$, with the duration of loss computation defined as $T_{1:loss}$, resulting in the graph encoders cross-entropy loss $L_{c}^{G}$ being calculated as follows:

(19) $$L_{c}^{G}=-\frac{1}{T_{loss}}\sum_{t=T_{1}}^{T_{loss}}\sum_{i\in G}P_{i}^{G}\left(t\right)log\left({\hat{P}}_{i}^{G}\left(t\right)\right)$$

Additionally, this study employs an exponential smoothing $L_{1}$ loss for the location encoder, defined as follows:

(20) $$L_{r}^{G}=\frac{1}{T_{loss}}\sum_{t=T_{1}}^{T_{loss}}\sum_{i\in G}{Smooth}_{L_{1}}\left(P_{i}^{G}\left(t\right),{\hat{P}}_{i}^{G}\left(t\right)\right)\times e^{\frac{T_{loss}-t+1}{\mu }}$$

Furthermore, this study introduces an exponential penalty term, denoted as $e^{\frac{T_{loss}-t+1}{\mu }}$, defined to guide the model to focus more on the predictions at earlier time steps. This focus is crucial as the accuracy of early trajectory predictions significantly influences subsequent trajectories. The hyperparameter μ is employed to control the intensity of this penalty term.

### 3.7. Generation of Multiple Trajectories

To generate diverse probabilistic trajectory distributions, we adopt various beam search strategies outlined in [29]. We define $B_{t-1}$ as the set of beams at time t−1, each containing *K* decoded trajectories. Each trajectory consists of a sequence of position indexes, each representing a potential path from the start to the current time step. Let $M_{t-1}^{k}=({\hat{y}}_{1}^{k},\ldots ,\;{\hat{y}}_{t-1}^{k},\;k\in \left[1,\;K\right]$) represent the k-th trajectory at time t−1, where ${\hat{y}}_{t}^{k}$ is the location-specific index in the scale graph G and $P_{t-1}^{k}$ is the cumulative logarithmic probability of the k-th trajectory from the start to time t−1. The probability distribution at the current time step *t* is computed based on the past trajectory $M_{t-1}^{k}$. $C_{t}^{k}$ computed from Equation (13), which describes the probability of predicting time step t from the past historical trajectory to predict the location of time step *t*. In Equation (13), $C_{t}$ is determined by the hidden state $H_{t}$, which itself is generated by the history trajectory $M_{t-1}^{K}$. $B_{t}$ is further expressed as $B_{t}={\mathrm{TOP}}_{K}\left(P_{t-1}^{k}+log\left(C_{t}^{k}\left(i\right)\right)+\xi \left(i\right)\;|\;k\in \left[1,K\right]\right)$ where $log\left(C_{t}^{k}\left(i\right)\right)$ is the logarithmic probability of predicting the next choice of the i-th position given $M_{t-1}^{K}$, and $\xi \left(i\right)$ is the diversity penalty, which reduces the chances of being chosen again, and increases trajectory diversity.

Specifically, the probability of $\left|V\right|\times K$ needs to be calculated for all nodes and beams in the scale graph G, where |V| is the number of nodes in G. In the flow of the graph encoder, each candidate output is an index of a graph node indicating the grid cell where the position of the next time step is located. In the position encoder process, each candidate output is a coordinate value indicating the offset of the position of the next time step relative to the center of the grid cell. At each time step, the algorithm selects the *K* highest probability out of all the candidate out-puts as the final prediction, and then uses them as inputs for the next time step to continue the search. In the position encoder process, the offsets are also added to the predicted grid cells to get the exact coordinates.

## 4. Experiment and Analysis

### 4.1. Dataset and Evaluation Metrics

#### 4.1.1. Benchmark Dataset

The Forking Path dataset is specifically designed for multifuture forecasting simulations. This dataset includes five scenarios from VIRAT/ActEV and four from ETH/UCY. It comprises 127 scenarios, each available in three 45-degree views and one top-down view. Each scene features several controlled pedestrians, each with an average of 5.9 future trajectories. The ActEV/VIRAT dataset, a public resource released by NIST in 2018, is intended for video activity detection research. It contains 455 videos captured at 30 frames per second and a resolution of 1080p, featuring 12 different scenes from various viewpoints. The ETH/UCY dataset includes five subscenes—ETH, HOTEL, ZARA1, ZARA2, and UNIV—encompassing a total of 1536 pedestrian trajectories. Trajectory data are converted into coordinate points in the world coordinate system, sampled at intervals of 0.4 s to form coordinate sequences. Additionally, all scenes are captured from a fixed top-down perspective. The Argoverse dataset is used for 3D tracking and motion forecasting in autonomous driving applications. It includes two subdatasets: 3D tracking and motion forecasting. The validation set video within the 3D tracking dataset is captured using the onboard front-center camera view.

#### 4.1.2. Multifuture Evaluation Metrics

To evaluate population multifuture trajectory predictions, we adopt the definition of the multifuture trajectory prediction task as described in [15], which involves generating the 20 most likely predictions (K=20) for each data sample. Predictions are assessed using the minimum Average Displacement Error ($minADE_{k}$) and the minimum Final Displacement Error ($minFDE_{k}$) across the *K* predictions. Additionally, the Percentage of Trajectory Usage (PTU) metric, proposed in [13], is utilized to gauge the overall performance of Pedestrian Multifuture Trajectory Predictions. This metric calculates the proportion of predicted trajectories utilized, assessed by $minADE_{k}$ and $minFDE_{k}$.

(21) $$\begin{array}{cc} minADE_{k} & =\frac{\sum_{i=1}^{N}\sum_{j=1}^{J}min_{k=1}^{K}\sum_{t=n+1}^{T}{∥Y_{t}^{ij}-{\hat{Y}}_{t}^{ik}∥}_{2}}{N\times (T-h)\times J}\\ minFDE_{k} & =\frac{\sum_{i=1}^{N}\sum_{j=1}^{J}{∥Y_{T}^{ij}-{\hat{Y}}_{T}^{ik}∥}_{2}}{N}\\ PTU & =\frac{\sum_{i=1}^{N}|{\hat{p}}_{i}\left|\setminus \right|Y_{i}|}{N} \end{array}$$

$minADE_{k}$: For each true trajectory *j* of test sample *i*, the one of the *K* predictions with the smallest distance from *j* is selected to compute the average displacement. $minFDE_{k}$: For each true trajectory *j* of test sample *i*, the coordinate that is closest to the endpoint coordinate value of trajectory *j* among the *K* predictions is selected as the minimum Final Displacement Error.

To quantitatively evaluate the safety and rationality of the predicted trajectories, we propose the Static Obstacle Collision Rate (SOCR) metric. It measures the percentage of predicted trajectory points that fall into non-walkable regions (e.g., walls, vehicles, vegetation) defined by the semantic segmentation map. A lower SOCR indicates higher safety and better adherence to static scene constraints. It is calculated as:

(22) $$SOCR=\frac{1}{N\times (T-n)\times K}\sum_{i=1}^{N}\sum_{k=1}^{K}\sum_{t=n+1}^{T}⊮\left(M\left({\hat{p}}_{t}^{i,k}\right)\in C_{obs}\right)\times 100\%$$

where *N* is the total number of pedestrians, *K* is the number of predicted modes (future trajectories), and T−n is the prediction horizon. ${\hat{p}}_{t}^{i,k}$ denotes the predicted position of the *k*-th trajectory for the *i*-th pedestrian at time *t*. M(·) is a function that maps the coordinate to a semantic class using the scene segmentation map, and $C_{obs}$ represents the set of obstacle classes (e.g., building, fence, pole, road, car). ⊮(·) is the indicator function, which equals 1 if the condition is met and 0 otherwise.

#### 4.1.3. Single-Future Evaluation Metrics

We use two metrics, Average Displacement Error (ADE) and Final Displacement Error (FDE), to evaluate our model, as follows:

(23) $$\begin{array}{cc} ADE & =\frac{\sum_{i=1}^{N}\sum_{t=n+1}^{T}{∥L_{t}^{i}-{\hat{L}}_{t}^{i}∥}_{2}}{N\times (T-n)}\\ FDE & =\frac{\sum_{i=1}^{N}{∥L_{T}^{i}-{\hat{L}}_{T}^{i}∥}_{2}}{N} \end{array}$$

where *N* denotes the number of pedestrians, *T* denotes the time step of the prediction, ${\hat{L}}_{t}^{i}$ is the trajectory generated by the model at time *t*, and $L_{t}^{i}$ is the trajectory of the ground truth at time *t*.

### 4.2. Implementation Details

We utilize the data processing method described in [9], starting by encoding input coordinates into a 32-dimensional vector via a fully connected layer, followed by ReLU activation. Scene semantic segmentation features are extracted using a pretrained DeepLabv3 model [51]. The MTP-STG model features a single-layer LSTM convolution as the backbone for both encoder and decoder, augmented by a graph attention mechanism that generates and aggregates information based on scale maps. Both spatial and temporal Transformers consist of encoding layers equipped with eight heads. Hyperparameter tuning on a scaled-down network determined the optimal learning rate to be 0.0015, using an Adam optimizer for model training. Training occurred in batches of 8 over 400 epochs, with each batch comprising around 256 pedestrians from various time windows, using an attention mask to speed up both training and inference processes. In the data augmentation module, adversarial trajectories are generated using the Targeted-FGSM attack method, iterating 10 times. Parameters in Equations (4) and (6) are set to ϵ=δ=0.1 and α=0.2. The trajectory prediction network utilizes a ConvLSTM architecture with an embedding size of 32, and encoder and decoder hidden layers each sized at 256. Hyperparameters in the multimodal trajectory prediction module are set at λ=1.0 and γ=0.2, and μ had a smoothing exponent of 5 for the calculations.

### 4.3. Quantitative Evaluation of MTP-STG

#### 4.3.1. Quantitative Analysis of Multifuture Trajectory Prediction

In this section, we evaluate the MTP-STG model using the Forking Paths Dataset, comparing it against baseline models including S-LSTM [8], S-GAN [9], Next [52], ST-MR [13], ST-AR [33], SimAug [12], Multiverse [15], TNT [53], MultiPath++ [54], and the AgentFormer [36]. Evaluation results for the minADE_20_ and minFDE_20_ metrics are presented across three perspectives: 45-degree, top-down, and full views. The PTU trajectory usage metric is exclusively evaluated for multifuture prediction models. The initial models are trained on the Forking Paths Dataset, with all models subsequently tested on this same dataset. From the results in Table 1, the MTP-STG model surpasses other baseline models across all evaluation metrics. Specifically, compared to the strong baseline ST-MR, MTP-STG reduces minADE_20_ by 2.3, 0.7, and 1.2 pixels across the metrics, and minFDE_20_ by 1.2, 1.7, and 2.1 pixels, while enhancing PTU trajectory usage by 0.5% and 0.4% respectively. To further demonstrate the competitiveness of our approach against newer methods, we compared it with prominent SOTA models: AgentFormer, TNT, and MultiPath++. While these methods achieve impressive results on clean datasets by modeling social–temporal interactions or utilizing target-driven anchors, their performance drops slightly in our end-to-end setting (e.g., minADE_20_ All: 162.9 for MultiPath++ and 163.5 for AgentFormer vs. 161.6 for MTP-STG). This is because these models typically assume perfect historical trajectories and are sensitive to perception noise. In contrast, our MTP-STG utilizes the proposed Memory Storage Module to robustly handle the noise and fragmentation introduced by the upstream MOTR tracker. This superior performance is attributed to the MTP-STG model’s enhanced attention mechanism and multiscale graph structure, which effectively simulate interactions between pedestrians and their environment. Additionally, the memory graph dynamically records trajectory temporal information during decoding, correcting positions that violate temporal consistency, thereby enhancing trajectory smoothness and rationality.

#### 4.3.2. Quantitative Analysis of Single Future Trajectory Prediction

Our experimental evaluation is based on the single future trajectory metrics outlined in [13,15], utilizing VIRAT/ActEV in conjunction with Argoverse as the evaluation datasets for pedestrian single future trajectory prediction. Consistent with previous studies, our evaluation observes a time step of 3.2 s (8 frames) and a prediction length of 4.8 s. The experimental results are presented in Table 2. The MTP-STG model, as proposed in this study, enhances performance on the ADE and FDE metrics compared to baseline models such as S-LSTM [8], S-GAN [9], Next [52], ST-MR [13], ST-AR [33], SimAug [12], and Multiverse [15]. This suggests that the MTP-STG model offers greater stability for both multifuture simulation and single future trajectory prediction in real-world scenarios.

#### 4.3.3. Evaluation of Trajectory Rationality and Safety

**Figure 6:**
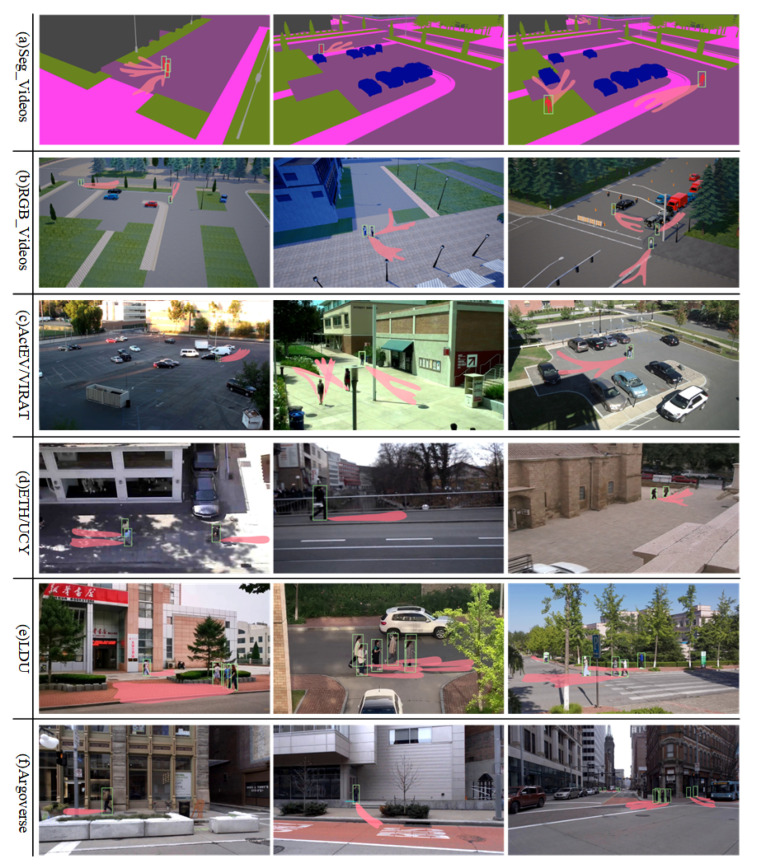
Heatmap visualization of Pedestrian Multifuture Trajectory Prediction under different benchmark datasets.

Beyond standard displacement metrics, evaluating the rationality and safety of predicted trajectories is crucial for real-world applications. We focus on two aspects: collision avoidance and adherence to social norms. We introduce the Static Obstacle Collision Rate (SOCR) to quantify safety. SOCR measures the percentage of predicted trajectory points that fall into non-walkable areas (e.g., walls, parked vehicles, vegetation) defined by the semantic segmentation maps. As shown in Table 3, our MTP-STG model significantly outperforms the baseline ST-MR. Thanks to the integration of the multiscale semantic grid graph, our model effectively “perceives” the environment, reducing the collision rate from 5.4% to 3.9%. In terms of social compliance, the generated trajectories should not only be collision-free but also follow walkable paths (e.g., sidewalks). Our qualitative results (discussed in Section 4.4.2 and Figure 6) demonstrate that MTP-STG predictions align strictly with sidewalk layouts, avoiding jaywalking in vehicle lanes. Furthermore, the high PTU scores (Table 1) indicate that our model generates diverse modes covering various plausible intentions, rather than collapsing to a single average path, thereby ensuring the diversity of the prediction.

### 4.4. Qualitative Evaluation of MTP-STG

#### 4.4.1. Pedestrian Detection Tracking Visualization

**Figure 5:**
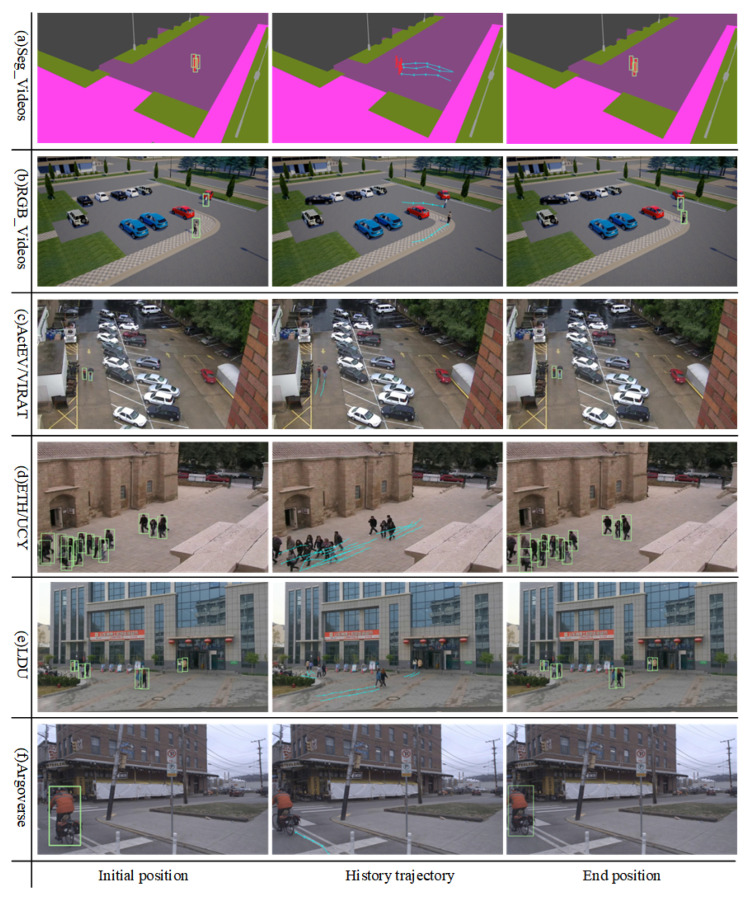
Effect of pedestrian detection performance of MOTR detector under different benchmark datasets.

In this paper, we visualize the performance of the MOTR detector across various benchmark datasets; Figure 5 illustrates the paths taken by pedestrians within 3.2 s of observation. The figure demonstrates that the Transformer-based MOTR detector accurately identifies pedestrians across various test scenarios in different benchmark datasets. It effectively extracts pedestrian features at multiple scales, handles complex occlusions and interactions, and maintains high detection accuracy and frame rates in densely crowded scenarios, thereby preventing trajectory loss.

#### 4.4.2. Multifuture Trajectory Heat Map Visualization

We also conduct trajectory prediction heatmap visualizations for each dataset to further analyze the semantic interpretability of the proposed MTP-STG model. Figure 6 displays the heatmap of Pedestrian Multifuture Trajectory Predictions for three different scenes in each dataset, generated by the GAT decoder on a 2D lattice grid. The figure illustrates that the MTP-STG model accurately predicts the intensity of pedestrian multifuture trajectories across all datasets. This accuracy stems from using simulation data for adversarial enhancement during training and employing a spatio-temporal graphical attention network to capture environmental details and self-assign attention weights to pedestrians.

#### 4.4.3. Multifuture Trajectory Prediction Visualization

**Figure 7:**
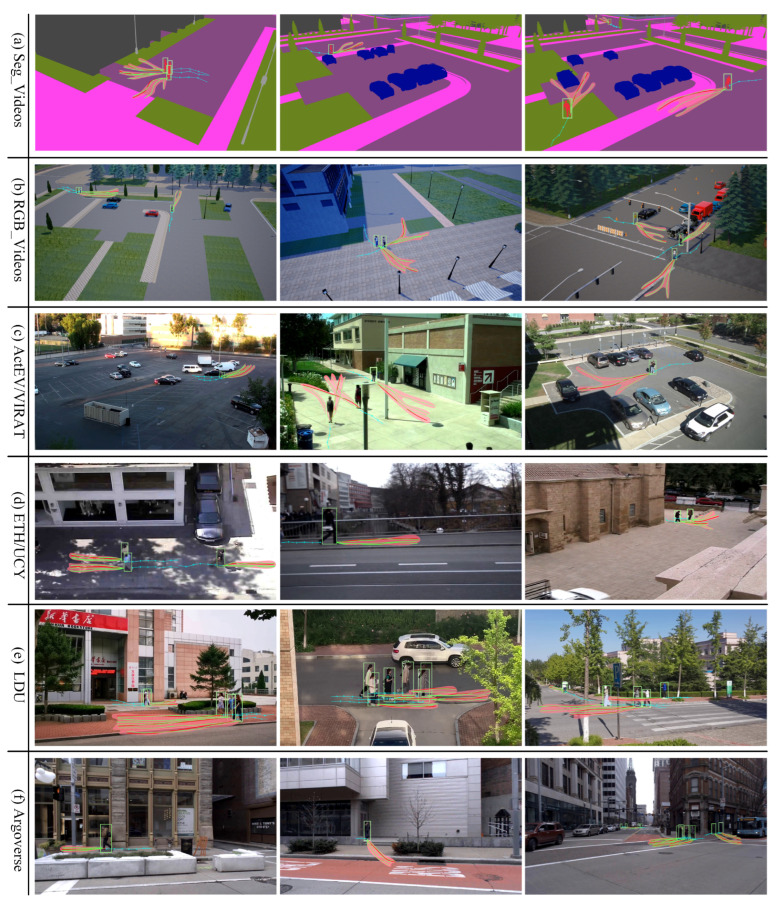
Qualitative visualization results of the MTP-STG model on the benchmark dataset. The top 3 highest rated possible trajectories were selected for visualization (K = 3).

Qualitative analysis of benchmark datasets. Figure 7 illustrates the predictive tracking performance of the MTP-STG model on pedestrian multifuture trajectories across various benchmark datasets. In Figure 7, blue line segments represent observed walking trajectories of the crowd; light red indicates the crowd’s multimodal trajectory locations output by the scale-map decoder, shown as a heatmap; green lines depict predicted future walking trajectories; red lines show actual future walking trajectories; and light green highlights pedestrian detection frames calibrated by the MOTR detector. The visualization results demonstrate the efficacy of our multifuture trajectory prediction model in forecasting crowd movements. In the third scenario of SegVideos semantic segmentation, the model effectively gathers environmental semantic information to navigate around stationary vehicles. In the Rgbvideos simulation, the MTP-STG model effectively predicts multimodal trajectories for pedestrians approaching each other, forecasting three potential paths. In the third scenario of the VIRAT/ActEV dataset, the model accurately tracks pedestrians carrying suitcases and predicts three potential future directions. In the LDU dataset with high crowd density, the DETR architecture and tracklet trajectory-aware label assignment enhance the MOTR detector’s ability to discern pedestrian appearances and locations for precise cross-frame matching. The location decoder outputs corrected multifuture trajectories, enhancing accuracy in crowded environments.

**Figure 8:**
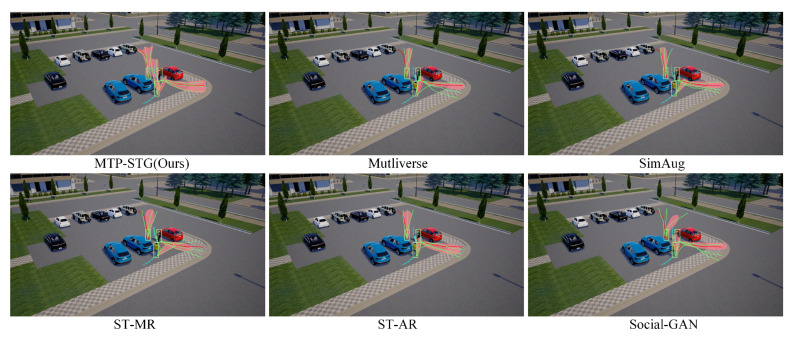
Comparison of qualitative results between MTP-STG and each baseline model on the benchmark dataset.

Qualitative analysis of the baseline models. Figure 8 displays the visualization results of the MTP-STG model compared to each baseline model on the Forking Path dataset. The MTP-STG model accurately predicts trajectories that align with the actual value distribution, avoiding collisions with other objects. Examples demonstrate that our model effectively makes informed decisions using pedestrian detections and historical trajectory data processed by a spatio-temporal graph attention network. Additionally, while the baseline models often exhibit temporal inconsistencies, the MTP-STG, leveraging a Memory Storage Module, maintains consistent trajectory predictions over time.

### 4.5. Ablation Study and Efficiency Analysis

#### 4.5.1. Ablations of Key Components

To systematically evaluate the contribution of each module in our MTP-STG framework, we conducted a comprehensive ablation study on the validation set. As shown in Table 4, we analyze six variants by removing specific components: Multiview Simulation Data (Sim), View Selection (View), Adversarial Attack (Adv), Multiscale Graph (MSG), Memory Module (Mem), Location Decoder (Dec), and Exponential Smooth L1 Loss (Loss). We did not include the $L_{1}$ exponential smoothing design in our tests for multiple future trajectory prediction, as the exponential loss is specifically tailored to enhance single future prediction during model training.

Table 4 presents the systematic ablation study results for multiple future trajectory prediction, while Table 5 details those for single future trajectory prediction. The tables demonstrate that the full MTP-STG model attains optimal accuracy across both tasks. This performance is achieved by effectively utilizing simulation data, learning from inconsistent viewpoints, and generating adversarial trajectories. Specifically, setting randomized perturbations with δ=0.1 and integrating random search viewpoint trajectories through the Mixup convex function [43] allows the model to adeptly handle fine-grained noises introduced by varying lighting conditions, scene textures, and camera sensors. In our studies, setting δ to 0 increased errors between sequence frames, thereby raising the computational error rate in the classification loss function (Equation (3)). Conversely, the random perturbation setting of δ=0.1 is specifically designed to minimize errors arising from data uncertainty. Furthermore, removing the adversarial network (without Adversarial Attack) causes accuracy to diminish. This decline is attributed to the GAN network’s ability to expand data from original viewpoint labels, reducing interference from varying backgrounds and mitigating overfitting.

For multifuture trajectory prediction, Table 4 indicates that the position decoder has the most significant impact on overall accuracy, followed by the multiscale graph and the Memory Storage Module. The position decoder computes offset coordinates for each node based on the graph encoder’s output, enabling precise position predictions and preventing restrictions to grid cell boundaries. The multiscale graph allows the model to adapt to varying levels of detail, facilitating decision making based on the pedestrian’s surroundings. Crucially, the Memory Storage Module functions as a trajectory smoothing algorithm. As indicated by the minFDE increase in Table 4 (w/o Memory Module), removing this component leads to temporal inconsistencies. Qualitatively, without the memory module, early-time predictions often exhibit high-frequency jitter (“zigzag” patterns). By conditioning predictions on historical embeddings, the memory module mitigates conflicts between spatial and temporal information, ensuring the generated paths are smooth and kinematically plausible.

Exponential smooth $L_{1}$ loss. Unlike multiple future trajectory prediction where diversity is prioritized, the $L_{1}$ exponential smoothing design is specifically employed to enhance the accuracy of single future trajectory prediction (Table 5). It emphasizes earlier data in the sequence, which influences overall performance. In Equation (20), different values of μ were tested. The hyperparameter μ regulates the strength of the penalty term, with selected values of +∞, 15, 10, and 5 (+∞ corresponds to using only smooth $L_{1}$ loss). The results in Table 6 indicate that the MTP-STG model achieves optimal performance when μ=5. On the ActEV/VIRAT dataset, ADE is reduced by 0.63 and FDE by 1.91% compared to models without $L_{1}$ exponential smoothing. These results suggest that selecting an appropriate μ value effectively balances the penalty term and loss function, thereby enhancing the accuracy of single future trajectory prediction.

**Figure 9:**
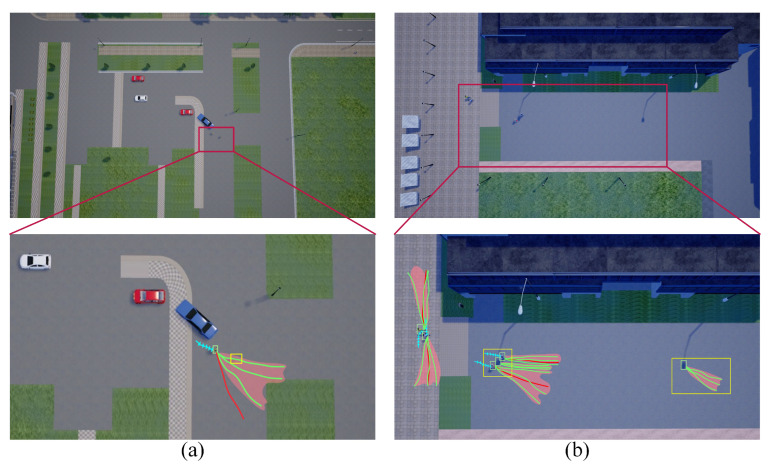
Example of limitations of our model. In scenario (**a**), missed detection leads to the failure of crowd trajectory prediction. In scenario (**b**), the model misidentifies the street lamp as a pedestrian, resulting in incorrect predictions.

Algorithmic limitations. We present examples of prediction failures in Figure 9, where yellow boxes highlight scenarios of missed and incorrect detections. In scenario (a), a missed detection leads to a failure in predicting crowd trajectories. In scenario (b), the model incorrectly identifies streetlights as pedestrians, resulting in erroneous predictions. Although top-view trajectories are incorporated in training, dynamic detection remains insensitive due to the small size of targets in this view. Additionally, the failure rate increases in scenes with significant lighting changes, indicating a need to enhance detection of small targets under varying lighting conditions. Furthermore, our model occasionally fails to accurately perceive pedestrian walking speeds, predicting trajectories longer than actual distances. Future work could improve performance by optimizing the prediction network loss function or diversifying the final position prediction approaches.

#### 4.5.2. Efficiency and Computational Cost

To evaluate the feasibility of MTP-STG for real-world deployment, particularly in intelligent transportation systems (ITS) and autonomous driving, we analyzed the model’s computational complexity and inference speed. All efficiency experiments were conducted on a workstation equipped with an NVIDIA GeForce RTX A6000 GPU and an Intel Xeon CPU. Table 7 presents the comparison of model parameters, inference time, and Frames Per Second (FPS) between our proposed MTP-STG and representative baseline methods. Although the introduction of the multiscale graph structure and the Memory Storage Module increases the number of parameters compared to lightweight LSTM-based models (e.g., S-LSTM), our method maintains a competitive inference speed. Specifically, MTP-STG achieves an inference speed of approximately 38.4 ms per frame, corresponding to 26 FPS. This efficiency is primarily attributed to the parallel computation capabilities of the Transformer architecture and the matrix-based retrieval of the memory module, which avoids the sequential bottlenecks typical of RNNs. To further assess practicality, we estimate the computational cost to be approximately 15.6 GFLOPs per frame. Crucially, for real-world ITS and autonomous driving applications, our framework supports an online/offline separation strategy. The semantic feature extraction for static scene elements (e.g., roads, buildings) can be computed offline and cached as a background feature map. Only the pedestrian detection (MOTR) and trajectory generation components require online inference. This decoupling significantly reduces the real-time computational burden, making MTP-STG highly practical for deployment on edge devices in smart city infrastructures.

## 5. Discussion and Conclusions

This paper explores the integrated framework of multitarget tracking and multifuture trajectory prediction for crowds. We have refined the MOTR detection tracker to enable end-to-end monitoring, leveraging the DETR architecture and automatic tracklet trajectory-aware label assignment to handle variations in pedestrian appearance. For trajectory prediction, we encode historical states into a pedestrian matrix and process them through a Spatio-Temporal Graph Transformer, which features a multiscale graph structure to capture both local and global context. Additionally, a Memory Storage Module was introduced to ensure temporal consistency and smooth trajectory generation.

Crucially, our framework demonstrates significant robustness under harsh real-world conditions, such as severe occlusion and extreme congestion. Our analysis indicates that the query-based mechanism in MOTR effectively retains target identity during temporary occlusions, while the memory module leverages historical embeddings to bridge gaps in visual data. Furthermore, in high-density crowd scenarios (e.g., LDU dataset), the proposed Spatio-Temporal Graph Attention mechanism explicitly models complex neighbor interactions. This allows the model to generate diverse, collision-free paths without collapsing into a single average trajectory, ensuring safety and rationality in congested environments. Beyond ground-level surveillance, the proposed MTP-STG framework exhibits inherent adaptability to broader Remote Sensing applications, particularly in aerial surveillance and smart city monitoring. Our Multiview Data Augmentation specifically trains the model to be robust across varying camera pitch angles (45° to 90°), making it highly suitable for the dynamic perspectives of Unmanned Aerial Vehicles (UAVs). Moreover, the Multiscale Grid Graph can be extended to incorporate high-resolution satellite imagery or GIS data. By aligning pedestrian tracking with these overhead semantic maps, MTP-STG holds the potential to predict crowd flows at a city scale, aiding in urban planning and emergency response.

Experiments on benchmark datasets demonstrate that our proposed MTP-STG model achieves state-of-the-art performance. The integration of end-to-end tracking with interaction-aware prediction offers a promising solution for intelligent transportation systems, autonomous surveillance, and future smart city infrastructures.

## Figures and Tables

**Table 1:** Quantitative evaluation of multifuture trajectory prediction.

Method	${\mathrm{minADE}}_{20↓}$				${\mathrm{minFDE}}_{20↓}$			
	45°	Top-Down	All	${\mathrm{PTU}}_{↑}$	45°	Top-Down	All	${\mathrm{PTU}}_{↑}$
S-LSTM	204.2	188.4	198.6	N/A	385.4	354.1	383.5	N/A
S-GAN	198.4	185.9	192.7	43.7%	367.5	348.5	369.8	43.6%
Next	184.5	177.3	182.6	N/A	355.2	350.6	363.3	N/A
SimAug	178.4	171.8	174.3	45.8%	347.8	339.2	351.7	44.3%
Multiverse	168.9	157.7	166.1	47.4%	333.8	316.5	329.5	45.7%
ST-AR	170.3	161.3	169.2	46.2%	322.3	317.9	330.6	45.8%
ST-MR	165.5	154.5	162.8	48.6%	318.9	302.5	314.8	50.8%
AgentFormer	166.2	155.1	163.5	48.1%	320.5	305.4	316.2	50.1%
TNT	167.5	156.4	164.8	47.5%	322.1	308.2	319.5	49.5%
MultiPath++	165.8	154.6	162.9	48.4%	319.4	304.1	315.8	50.5%
MTP-STG	163.2	153.8	161.6	48.9%	317.7	300.8	312.7	51.2%
${\text{MTP-STG}}^{*}$	159.4	151.9	159.4	49.3%	315.4	298.7	310.1	52.4%

Note: ↓ indicates lower values are better; ↑ indicates higher values are better. ${\text{MTP-STG}}^{*}$ indicates the MTP-STG model further fine-tuned on real-world scenario datasets.

**Table 2:** Quantitative evaluation of single future trajectory prediction.

Method	ActEV/VIRAT		Argoverse	
	ADE	FDE	ADE	FDE
S-LSTM	23.10	44.27	N/A	N/A
S-GAN	30.42	60.70	N/A	N/A
Next	19.78	42.43	N/A	N/A
ST-AR	22.76	38.89	70.4	179.2
ST-MR	18.58	36.08	68.5	177.3
SimAug	21.73	42.22	67.9	175.6
Multiverse	18.51	35.84	69.1	183.9
MTP-STG	18.54	35.33	67.3	174.3
${\text{MTP-STG}}^{*}$	17.41	35.25	65.8	173.2

${\text{MTP-STG}}^{*}$ indicates the MTP-STG model further fine-tuned on real-world scenario datasets.

**Table 3:** Comparison of Static Obstacle Collision Rate (SOCR) on the VIRAT/ActEV dataset.

Method	Semantic Input	SOCR (%) ↓
S-LSTM	No	9.2
ST-AR	No	8.7
Multiverse	Yes	7.4
SimAug	Yes	6.2
ST-MR	Yes	5.4
MTP-STG (Ours)	Yes	3.9

Note: ↓ indicates lower values are better.

**Table 4:** Ablation study of the key design in multifuture prediction models.

Model Variant	Components						minADE_20_ ↓				minFDE_20_ ↓			
	Sim	View	Adv	MSG	Mem	Dec	45°	Top	All	${\mathrm{PTU}}_{↑}$	45°	Top	All	${\mathrm{PTU}}_{↑}$
**MTP-STG (Full)**	✓	✓	✓	✓	✓	✓	**163.2**	**153.8**	**161.6**	**48.9%**	**317.7**	**300.8**	**312.7**	**51.2%**
w/o View Selection	✓	×	✓	✓	✓	✓	165.7	157.4	167.1	45.3%	329.8	318.6	330.4	46.0%
w/o Adversarial Attack	✓	✓	×	✓	✓	✓	167.4	160.7	170.9	41.2%	330.6	321.4	332.5	44.7%
w/o Multiscale Graph	✓	✓	✓	×	✓	✓	177.6	188.4	169.5	43.4%	339.4	320.7	337.2	42.5%
w/o Memory Module	✓	✓	✓	✓	×	✓	169.8	152.9	167.4	46.1%	321.6	306.5	326.8	46.3%
w/o Location Decoder	✓	✓	✓	✓	✓	×	249.7	228.4	257.3	30.7%	398.2	410.8	405.6	29.6%
w/o Simulation Data	×	✓	✓	✓	✓	✓	169.1	162.5	173.5	39.5%	334.2	323.9	335.8	43.1%

Note: ↓ indicates lower values are better; ↑ indicates higher values are better.

**Table 5:** Ablation Study of the Key Design in Single Future Prediction Models.

Method	ActEV/VIRAT		Argoverse	
	ADE ↓	FDE ↓	ADE ↓	FDE ↓
w/o View Selection	21.34	40.80	70.7	180.4
w/o Adversarial Attack	20.18	39.55	69.7	181.9
w/o Multiscale Graph	21.13	38.26	71.8	181.3
w/o Memory Module	18.97	36.73	69.6	177.5
w/o Location Decoder	40.78	59.32	94.2	230.6
w/o Smooth $L_{1}$ Loss	19.37	37.24	69.3	176.2
MTP-STG Full Model		35.33	67.3	174.7

Note: ↓ indicates lower values are better.

**Table 6:** Effect of different μ values on the accuracy of single future prediction.

Value of μ	ActEV/VIRAT		Argoverse	
	ADE	FDE	ADE	FDE
μ=+∞	19.37	37.24	69.3	176.2
μ=15	19.26	36.79	68.6	175.9
μ=10	18.87	36.24	67.8	174.8
μ=5	18.74	35.53	67.3	174.3

**Table 7:** Comparison of computational efficiency and model performance on the benchmark dataset.

Method	Inference Time (ms)	FPS	minADE_20_ ↓	minFDE_20_ ↓
S-LSTM	12.5	80	198.6	383.5
SimAug	32.1	31.2	174.3	351.7
Multiverse	37.4	26.7	166.1	329.5
ST-AR	36.6	27.3	169.2	330.6
ST-MR	37.7	26.5	162.8	314.8
MTP-STG (Ours)	38.4	26	161.6	312.7

Note: ↓ indicates lower values are better.

## Data Availability

The data supporting the findings of this study are available from the corresponding author upon reasonable request. Please contact us by email if you wish to obtain access to the data.
